# Plant circadian clock control of *Medicago truncatula* nodulation via regulation of nodule cysteine-rich peptides

**DOI:** 10.1093/jxb/erab526

**Published:** 2021-12-01

**Authors:** Mingkee Achom, Proyash Roy, Beatriz Lagunas, Emma Picot, Luke Richards, Roxanna Bonyadi-Pour, Alonso J Pardal, Laura Baxter, Bethany L Richmond, Nadine Aschauer, Eleanor M Fletcher, Monique Rowson, Joseph Blackwell, Charlotte Rich-Griffin, Kirankumar S Mysore, Jiangqi Wen, Sascha Ott, Isabelle A Carré, Miriam L Gifford

**Affiliations:** 1 School of Life Sciences, Gibbet Hill Road, University of Warwick, Coventry CV4 7AL, UK; 2 Department of Genetic Engineering & Biotechnology, University of Dhaka, Dhaka, Bangladesh; 3 Warwick Medical School, University of Warwick, Coventry CV4 7AL, UK; 4 School of Biological Sciences, University of Bristol, 24 Tyndall Avenue, Bristol BS8 1TQ, UK; 5 Wellcome Centre for Human Genetics, University of Oxford, Oxford OX3 7BN, UK; 6 Institute for Agricultural Biosciences, Oklahoma State University, Ardmore, OK 73401, USA; 7 Warwick Integrative Synthetic Biology Centre, University of Warwick, Coventry CV4 7AL, UK; 8 University of Glasgow, UK

**Keywords:** Circadian biology, *Medicago truncatula*, nitrogen fixation, nodulation, plant–environment interaction, symbiosis

## Abstract

Legumes house nitrogen-fixing endosymbiotic rhizobia in specialized polyploid cells within root nodules, which undergo tightly regulated metabolic activity. By carrying out expression analysis of transcripts over time in *Medicago truncatula* nodules, we found that the circadian clock enables coordinated control of metabolic and regulatory processes linked to nitrogen fixation. This involves the circadian clock-associated transcription factor LATE ELONGATED HYPOCOTYL (LHY), with *lhy* mutants being affected in nodulation. Rhythmic transcripts in root nodules include a subset of nodule-specific cysteine-rich peptides (NCRs) that have the LHY-bound conserved evening element in their promoters. Until now, studies have suggested that NCRs act to regulate bacteroid differentiation and keep the rhizobial population in check. However, these conclusions came from the study of a few members of this very large gene family that has complex diversified spatio-temporal expression. We suggest that rhythmic expression of NCRs may be important for temporal coordination of bacterial activity with the rhythms of the plant host, in order to ensure optimal symbiosis.

## Teaser

A nodule circadian clock involving the gene *Late Elongated Hypocotyl*and rhythmic expression of nodule-specific cysteine-rich peptides coordinates waves of metabolic and regulatory activity during symbiosis.

## Introduction

In plants, animals, and microbes, many aspects of physiology, metabolism, and development exhibit 24 h rhythmicity controlled by a circadian clock. Under natural day–night conditions, the circadian clock is synchronized to light–dark and temperature cycles and enables anticipation of predictable daily changes in the environment. Rhythmicity is particularly pervasive in plants. In the model plant *Arabidopsis thaliana*, ~30% of genes are expressed rhythmically in constant light, and up to 90% are under at least some cycling environmental conditions (Michael *et al.*, 2008). Many aspects of metabolism are rhythmic, including photosynthetic carbon (C) assimilation, and nitrogen (N) and sulfur metabolism (Harmer *et al.*, 2000), and appropriate timing of starch utilization is known to ensure optimal growth (Graf *et al.*, 2010). The circadian clock also impacts plant productivity and health by modulating interactions with microorganisms. Plants show different levels of resistance to fungal and bacterial pathogens depending on the time of infection (Bhardwaj *et al.*, 2011; Zhang *et al.*, 2013; Ingle *et al.*, 2015; Lu *et al.*, 2017), and bacterial infections have been found to alter plant circadian regulation in order to attenuate immune responses (de Leone *et al.*, 2020). Plant circadian rhythms also influence the composition of rhizosphere microbial communities, and impeded circadian clock function in the plant host results in the recruitment of a different root microbiome, with consequences for plant health (Staley *et al.*, 2017; Hubbard *et al.*, 2018).

The mechanism of the plant circadian clock has been studied extensively and shown to consist of a small gene network comprising multiple transcriptional feedback loops (McClung, 2019). In *A. thaliana*, a pair of closely related MYB transcription factors, late elongated hypocotyl (LHY) and circadian clock associated 1 (CCA1), are expressed in the morning and act to repress the expression of other clock components, by binding to a DNA sequence motif in their promoters known as the evening element or EE (AAATATCT/AGATATTT) (Harmer *et al.*, 2000). As *LHY/CCA1* expression declines, a set of pseudo-response regulators (*PRR9*, *PRR7*, *PRR5*, and *PRR1*, also known as *TOC1*) are expressed as sequential waves during the day and early evening, and act to repress expression of *LHY* and *CCA1* till the following dawn. A third set of proteins, composed of LUX ARRYTHMO (LUX), EARLY FLOWERING (ELF3), and ELF4, is expressed at dusk and forms an ‘evening complex’. There is evidence that a similar mechanism operates in roots, although whereas the leaf circadian clock is primarily synchronized to diurnal light–dark cycles, the root circadian clock is thought to be entrained by shoot-derived signals (James *et al.*, 2008; Takahashi *et al.*, 2015; Bordage *et al.*, 2016). Circadian clock components are conserved in both monocot and dicot crops, and have been linked to important agronomic traits including growth and flowering time (Bendix *et al.*, 2015). Homologues of *A. thaliana* circadian clock genes have been identified in most legumes including soybean (*Glycine max*), cow pea (*Vigna unguiculata*), and garden pea (*Pisum sativum*) (Weller and Ortega, 2015; Weiss *et al.*, 2018; Li *et al.*, 2019; Wang *et al.*, 2020). In *A. thaliana*, LHY and CCA1 function as transcriptional repressors (Nagel *et al.*, 2015; Adams *et al.*, 2018) and have a largely redundant function in the central oscillator of *A. thaliana*. A single orthologue of these proteins is present in *Medicago truncatula*, termed *MtLHY* (Hecht *et al.*, 2005). Recent work has suggested that *LHY* is involved in nodulation (Kong *et al.*, 2020), although the mechanism by which this occurs was not characterized.

Altered function of the soybean circadian clock through overexpression of a light signalling component has been seen to lead to grain yield increases (Preuss *et al.*, 2012). However, there is a lack of information about the impact of the circadian clock on legume symbioses with N-fixing rhizobia. This is important, because this symbiosis contributes to the N nutrition of the plant which increases plant growth, reducing the need for synthetic N fertilizers while also improving soil health. During nodulation, rhizobia are accommodated in specialized root organs called nodules. Formation of nodules is initiated following recognition, by host plant LysM receptors, of Nod factors released from rhizobial bacteria. This leads to activation of calcium oscillations, then transcriptional responses that enable controlled cell division for nodule formation, and rhizobial entry via an infection thread. Within nodules, rhizobia inhabit an intracellular compartment derived from host cell membranes, called the symbiosome. They proliferate and differentiate into N-fixing bacteroids, which convert atmospheric di-nitrogen into a plant-accessible form such as ammonium that the host plant will incorporate into its own N metabolism. In exchange for the fixed N, the bacteria benefits from host-supplied C and other nutrients (Sprent and James, 2007; Maroti and Kondorosi, 2014). The evolution of nodulation in legumes has been greatly shaped by a whole-genome duplication event ~58 million years ago resulting in amplified, rearranged gene families and retention of paralogous genes (Young *et al.*, 2011). Prominent amongst these is the nodule cysteine-rich (NCR) gene family of small secreted peptides that are highly specific to nodules (Roy *et al.*, 2020). Except for some Aeschynomene species from the relatively ancient dalbergoid lineage, NCRs are exclusively found in the inverted repeat-lacking clade (IRLC) of legumes which includes the model plant *M. truncatula* and many agriculturally important crops such as alfalfa, clovers, lentils, chickpea, garden pea, and fava beans (Czernic *et al.*, 2015). Only a few NCRs have been characterized in detail so far, but a picture is emerging of the importance of functional diversity for this gene family (Roy *et al.*, 2020). The diverse spatio-temporal expression profiles of NCRs (Guefrachi *et al.*, 2014; Nallu *et al.*, 2014; Roux *et al.*, 2014), high level of expression specificity across nodules (Mergaert *et al.*, 2003; Nallu *et al.*, 2013), and variation in amino acid sequence and isoelectric points (Kondorosi *et al.*, 2013) could enable this functional variation. Transcriptomic profiling shows that a subset of NCRs is regulated by N availability and by autoregulation of nodulation, suggesting an additional role for NCRs in controlling nodule development depending on cues from the environment (Lagunas *et al.*, 2019).

Here we show that in *M. truncatula* nodules, disrupted circadian rhythmicity through loss of function of the core circadian clock gene *LHY* results in reduced nodulation, suggesting that the circadian clock may impact on plant–rhizobia interactions in nodules. We investigate potential mechanisms through analysis of the rhythmic transcriptome in nodules and reveal circadian control of a subset of NCR genes through EE motifs in their promoters. We suggest that circadian regulation of NCR gene expression in nodules may play a role to ensure temporal coordination of bacterial activity with the rhythms of the plant host. Optimizing the timing of nodule-specific N fixation-regulatory peptides may allow improvement of N fixation without altering any aboveground circadian clock features. This may represent an interesting target for sustainable agriculture of legume crops.

## Materials and methods

### Plant materials and growth conditions

*Medicago truncatula* wild-type accession A17 in the Jemalong background was obtained from the IGER seed bank (http://www.igergru.ibers.aber.ac.uk). *Tnt1 M. truncatula* mutant lines for *LHY* (Medtr7g118330) in the R108 background were identified from the Noble Research Institute (https://medicago-mutant.noble.org/mutant/database.php) (Tadege, 2008) by querying the *LHY* coding region plus 200 bp upstream and downstream using a blastn search with default parameter settings (E-value cut-off 10^–6^); *Tnt1* lines were selected based on their E-values and percentage identity >95. Lines NF17115 (*lhy-1*) and NF16461 (*lhy-2*) were identified with insertions in the promoter region and the fifth exon, respectively (Fig. 2B).

Seeds were scarified with concentrated H_2_SO_4_, sterilized by treating with 7% sodium hypochlorite solution, then washed with sterile water. Seeds were sown on 1.5% phyto-agar plates, sealed using 3M Micropore™ tape, wrapped in foil then left at 4 °C for 72 h. Plates were susequently placed in a Sanyo MLR-352 growth chamber (25 °C) for 4 d before seedlings with a radicle length of >2 cm were transferred to FP11 pots containing sterilized perlite with a 1–2 cm layer of sterilized vermiculite on top. Pots were placed in a Sanyo 2279 growth cabinet with 12/12 h light/dark (12L:12D), irradiance of 200 μmol m^−2^ s^−1^, and temperature of 24 °C (day) and 21 °C (night). Pots were watered 2–3 times a week with modified Broughton and Dilworth (1971) nutrient solution (1 mM CaCl_2_, 1 mM KH_2_PO_4_, 75 µM FeNaEDTA, 1 mM MgSO_4_, 0.25 mM K_2_SO_4_, 6 µM MnSO_4_, 20 µM H_3_BO_3_, 1 µM ZnSO_4_, 0.5 µM CuSO_4_, 50 nM CoSO_4_, 0.1 µM Na_2_MoO_4_, adjusted to pH 6.5 with KOH). For plant growth for genotyping or seed bulking, germinated seedlings were transferred to FP9 pots containing F2 compost and plants were grown in a glasshouse compartment at 16/8 h light/dark, average irradiance of 200 μmol m^−2^ s^−1^, and temperature of 24 °C (day) and 21°C (night).

### *lhy* mutant characterization and rhythmic leaf movement assays

For phenotypic analysis, plants were grown under 12L:12D dark for 5 weeks, removed from pots, and photographed before measuring shoot and nodule weights. Individual nodule number was determined by counting mature nodules, and meristem number by counting individual branched lobes comprising mature nodules. Nodules were imaged using light microscopy. For rhythmic leaf movement (RLM) assays, plants were grown under 12L:12D for 10 d before transferring to constant light for imaging from above using time-lapse cameras (Brinno). Opening and closing of the first true leaf was monitored by measuring changes in visible leaf area using ImageJ software. Greyscale images were thresholded and converted to binary with leaves showing white on a dark background. White pixels were then quantified over time in regions of interest using the Integrated Density tool. The experiment was repeated three times, then data from all biological replicates combined. Baseline detrending was applied to the data, and periodicity for the remaining samples was analysed using FFT-NLLS in BioDare2 (Zielinski *et al.*, 2014).

### Rhizobial culture preparation and seedling inoculation for time course analysis

*Sinorhizobium meliloti* strain WSM1022 was grown on TY/Ca^2+^ plates (5 g l^–1^ tryptone, 3 g l^–1^ yeast extract, 6 mM CaCl_2_·2H_2_O, pH adjusted to 6.8–7.0) at 28 °C for 2 d. The bacteria were then spot-inoculated into 10 ml of liquid TY/Ca^2+^ medium and grown for ~24 h with gentle shaking at 28 °C. Rhizobial cells were harvested by centrifugation at 3200 *g* for 10 min, washed twice with sterile water, then resuspended in sterile water to an OD_600_=0.05. A 250 μl aliquot of freshly prepared rhizobial solution was used to inoculate each *M. truncatula* seedling the day after potting by pipetting onto the vermiculite layer in close proximity to plants.

### Sampling plants for transcriptomic analysis

For RNA-seq/quantitative PCR (qPCR) time-course analysis, after 40 d in 12L:12D, pots were transferred to constant light conditions at the same irradiance. At 0 h, and then every 3 h up to 48 h, plants were removed from pots, samples were pooled from 6–7 plants for each of three biological repeats, immediately flash-frozen, and stored at –80 °C. Nodules were picked from roots using tweezers, part of each root system without nodules was collected, and leaves were collected as 4–5 trifoliates. For measurement of NCR expression levels via RNA-seq in *lhy-1* and *lhy-2* versus R108, plants were sampled 1–2 h after dawn [Zeitgeiber time (ZT) 1–2]. Samples for qPCR analysis of *LHY* expression in mutants versus R108 (Fig. 2C) were taken at 07.30 h (morning) and 15.30 h (evening) into the light cycle.

### Genomic DNA extraction and PCR for *Tnt1* line genotyping

*Tnt1 M. truncatula* mutant lines were sterilized, germinated, and grown to maturity in a glasshouse compartment. Genomic DNA from a leaf sample from each of the plants was extracted using 5% Chelex suspension column binding and heat treatment (100 °C for 5 min), then diluted 1/10. Gene-specific primers *lhy-1*Fp (CTCAAAACATGGCGGCTTAC), *lhy-1*Fp (AGTGGCTGAGATTGGTTGTG), *lhy-2*Fp (AATGAACGATTTTAGCAGCGG), and *lhy-2*Rp (TTTGGCCGTATGCAAATGTAG) were designed based on the R108 sequence ~1000 bp away from the flanking sequence tag site for each of the *Tnt1* mutant inserts using Primer3. Gene-specific primers were used in combination with *Tnt1*-specific *Tnt1*-Fg (ACAGTGCTACCTCCTCTGGATG) and *Tnt1*-Rg1 (CAGTGAACGAGCAGAACCTGTG) primers for PCR genotyping (Cheng *et al.*, 2014; Veerappan *et al.*, 2014). MyRed Taq DNA polymerase (Bioline) was used in a reaction volume of 20 μl, utilizing touch-down PCR as described in Cheng *et al.* (2014).

### RNA extraction, RNA-seq, and qPCR analysis

Frozen plant tissue samples were finely ground using a mortar and pestle, then ~100 mg of each powdered sample was used for total RNA extraction followed by gDNA removal, using the Monarch® Total RNA Miniprep Kit. The quantity (>100 ng μl^–1^) and quality (RNA integrity >8.5) of RNA were determined using a Bioanalyzer 2100 RNA 6000 Pico Total RNA Kit (Agilent Technologies). Samples containing >5 μg of RNA in total were used for RNA-seq. mRNA library preparation, quality assessment, and sequencing (150 bp, unstranded, paired-end) were carried out by Novogene; mRNA libraries were prepared following the Illumina TruSeq™ RNA library preparation protocol, after rRNA had been removed using the Ribo-Zero kit.

For qPCR analysis, cDNA was prepared using the ProtoScript II First Strand cDNA Synthesis Kit from New England Biolabs (UK) Ltd. qPCR was performed with 20 µl reaction volumes using SYBR Green JumpStart Taq ReadyMix (Sigma-Aldrich) and 40 two-step amplification cycles (95 °C and 60 °C for 30 s and 60 s, respectively) in a 96-well Agilent Mx3005P real-time PCR machine. Primer pairs designed based on the borders of the second and third exons (F, CACAAAACAAAGAGAACGATGG; Rp, ATGGCTCCTGATTTGCACAG) were used for the quantification of *LHY* expression, normalized against the reference gene Mtβ-Tubulin Medtr7g089120 (Fp, TTTGCTCCTCTTACATCCCGTG; Rp, GCAGCACACATCATGTTTTTGG) which has been shown to be invariant in the conditions described (Walker, 2019). Data were analysed using the ΔC_t_ method, a derivation of the ΔΔC_t_ method (Livak and Schmittgen, 2001).

### Statistical analysis of transcriptomic levels

Raw sequence data in the form of a pair of fq.gz files with sequencing depth of at least 20 million reads per sample were processed using tools on the Galaxy EU server (usegalaxy.eu). First, the quality of raw sequencing data was analysed by FastQC (Andrews, 2010). Replicate 2 for 21 h and replicate 3 for 15 h were found to have poor quality data and were removed from analysis. Contaminating adapter sequences and poor quality sequences were removed by Trimmomatic v36.4 (Bolger *et al.*, 2014) with the following settings: slidingwindow: 4:20 and minlen: 40, and using phred33 quality scores. Next, these clean, trimmed, and paired reads were used to generate raw transcript read counts and transcripts per million (TPM) normalized read counts using Salmon quant v0.14.1 (Patro *et al.*, 2017) with *M. truncatula* reference transcript sequences (Mt4.0v1) downloaded from the Phytozome database (phytozome.jgi.doe.gov). Expression data for a total of 61 510 transcripts were generated. Read counts were further normalized as log2 transcripts per million (logTPM). To identify the diurnally oscillating transcripts, logTPM expression data were analysed using the R package MetaCycle v1.2.0 (Yang and Su, 2010) with the following settings: minper: 20, maxper: 28, cycMethod: LS (Lomb-Scargle).

Hierarchical clustering using the total within-cluster sum of square (elbow method) was performed in R using 1–Pearson’s correlation coefficient as a dissimilarity distance measure between normalized (mean centred and scaled by SD), oscillating genes. Enrichment analysis for processes was performed with the Bonferroni method of correction (*P*-values <0.05).

### Promoter motif presence and structure analysis

Promoter sequences of *M. truncatula* (Mt4.0v1) genes were retrieved from the *M. truncatula* genome database (http://www.medicagogenome.org). Promoter motif analysis was carried out using the MEME suite (Bailey *et al.*, 2015), and *de novo* motif discovery runs were performed on either strand of unaligned 500 bp upstream sequence with motif width of 12 bp. We subsequently also queried 200 bp and 1000 bp of each NCR promoter, finding that motifs were clustered within the 500 bp region; this location is consistent with findings from Nallu *et al.* (2013). Conserved motifs were selected based on bit size (range from 0 to 2), positional bias (*P-*value <0.05), and with an E-value <0.001 (Supplementary Fig. S1).

The *A. thaliana* LHY-binding site position weight matrix (PWM) was downloaded from the JASPAR database and a PWM was produced for the evening element-related (EER) motif using expression of the promoters of 166 *M. truncatula* genes in roots 2–6 h after dawn (Lagunas *et al.*, 2019). The presence of motifs in *M. truncatula* in 500 bp promoter upstream sequences was determined using Find Individual Motif Occurrence (FIMO) (Grant *et al.*, 2011), with hits with a *P*-value <1e^–4^ indicating presence of the motif. Enrichment was determined using a hypergeometric test in R.

### Multiple sequence alignment of NCR promoters

Promoter sequences were aligned using MAFFT (Multiple Alignment using Fast Fourier Transform) tools at EBI (European Bioinformatics Institute) (Katoh *et al.*, 2019). The promoters of eight cycling NCR genes were aligned using a hidden Markov Model (HMM), selected based on the occurrence of all the three motif sites. For optimal alignment and representation of motif conservation, we ran algorithms with a parameter setting of gap lowest open penalty 1 allowed in MAFFT, gap extension 0.5, and an iteration of 100 runs; due to the diverse nature of the NCR sequences (Branca *et al.*, 2011), gaps were required to generate the best local and global alignment (Mount, 2008). The aligned sequences were then visualized in Genious v.11.0.2 (https://www.geneious.com).

### Transcription factor search and orthologue identification

Tomtom (Gupta *et al.*, 2007) was used to search for PWM query motifs against the *A. thaliana* PBM db (Franco-Zorrilla *et al.*, 2014), DAP motifs (O’Malley, 2016), and JASPAR plants 2018 databases of known transcription factor-binding sites. For othologous gene identification in *M. truncatula*, we used two methods. Firstly, reciprocal BLASTp was performed with the NCBI blast suite; alignment score, percentage query coverage, and expect value were determined for forward (*A. thaliana* protein query to *M. truncatula* protein database) and reciprocal (top *M. truncatula* hit to *A. thaliana* database) queries (Altschul *et al.*, 1990) with highest scoring pairs designated orthologues. Secondly, a Smith–Watermann (SW) alignment homologue search was carried out using the Phytozome v12.1.6 database. Different homologues of the same gene are marked as a/b/c, based on their level of similarity to the *A. thaliana* orthologue, with ‘a’ being the highest.

### Promoter phylogenetic tree reconstruction

DNA sequences of 700 NCRs with their upstream region were aligned using MPI-based MAFFT v7.3 for large sequences (Katoh *et al.*, 2019). Maximum-likelihood (ML) analyses and search for the best-scoring tree were performed using RAxML v.8.2.10 with rapid bootstrapping of 100 replica runs. The substitution model of generalized time reversal (GTR) and the gamma model of rate heterogeneity were used. The best resulting ML tree for DNA alignments was used for visualization with FigTree v.1.4.4. The presence of the EE (AGATATTT), EER (AGACATTT), or both AGAC/TATTT in the promoter for each NCR was then highlighted manually with colours on FigTree.

### CCA1/LHY phylogenetic tree reconstruction

Circadian clock gene homologues were initially identified via reciprocal BLASTp performed with the NCBI blasp suite of *A. thaliana* protein sequences against sequences in the National Center for Biotechnology Information (NCBI). Hits were sorted primarily by maximum bitscore score followed by E-value. Homology was also assessed using the SW alignment homologue search with the Phytozome v12.1.6 database. Evolutionary history was inferred using the ML method and JTT matrix-based model (Jones *et al.*, 1992). Initial trees for the heuristic search were obtained by applying Neighbor–Joining and BioNJ algorithms to a matrix of pairwise distances estimated using the JTT model. Trees with superior log likelihoods are shown and are visualized using the interactive tree of life v4 (Letunic and Bork, 2019).

## Results

### Gene expression within *Medicago* nodules and roots shows the presence of functional belowground circadian clocks

**Fig. 1.**
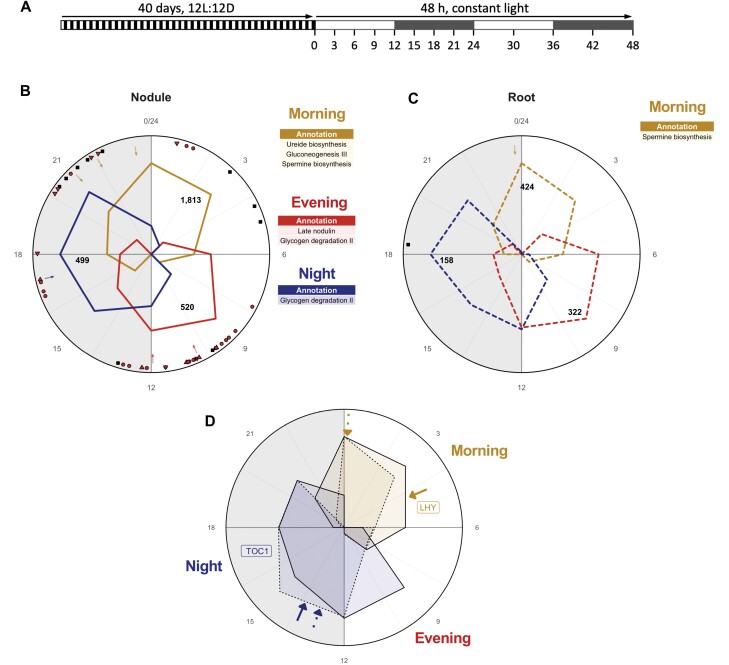
Oscillating expression of the belowground tissue circadian clocks. (A) Experimental design for 48h time course with sampling points. (B) Nodule and (C) root normalized mean transcription levels of clusters of rhythmic transcripts that peak at different times of the day (gold indicates a morning peak, red an evening peak, and blue a peak at night). Enriched biological pathways in each cluster, protein motifs, and GO terms have been listed in the coloured boxes; see Dataset S4 at Dryad for process details. Arrows indicate the mean MetaCycle predicted peak of transcripts associated with these processes. Black and red symbols indicate the peak transcription of a rhythmically expressed NCR transcript; a red circle indicates that the NCR gene promoter contains both an EE and EER, a triangle indicates that it contains an EE, an inverted triangle that it contains an EER, and a black square indicates that it has neither motif. (D) Expression profiles of *lhy* (gold) and *toc1* (navy blue) in nodules (solid line; see Dataset S1 at Dryad) and roots (dotted line; see Dataset S2 at Dryad). Arrows indicate the time of the peak as predicted by MetaCycle.

In order to ask which processes involved in belowground organ function might be under circadian regulation, we carried out a time course analysis of the rhythmic transcriptome. Plants were grown under diurnal light–dark cycles (12L:12D) for 40 d in order to entrain their circadian clocks, then transferred to constant light to test for persistence of rhythms in the absence of environmental time cues. Nodules and roots were sampled every 3 h for the first 24 h in constant light, then every 6 h for another 24 h (Fig. 1A), and changes in gene expression were analysed by RNA-seq. For nodules, after normalization and mapping of reads to the *M. truncatula* genome (4.0v1), we were able to determine expression levels for 61 510 transcripts out of the 62 319 protein-coding transcripts in the genome (98.7%). In roots, expression levels were determined for 84.6% of transcripts. We then used Metacycle analysis (Wu *et al.*, 2016) to identify transcripts that oscillate with a period of ~24 h (Fig. 1B, C). This identified 2832transcripts with rhythmic behaviour in constant light in nodules (~5% of the transcriptome) and 904 transcripts with rhythmic behaviour in constant light in roots (~1.3% of the transcriptome) (Supplementary Fig. S2; Dataset S1, S2 at Dryad; Achom *et al.*, 2022).

To examine the circadian clock regulation of belowground rhythms, we first profiled the expression patterns of circadian clock-associated genes in nodules and roots. Putative orthologues of all *A. thaliana* circadian clock genes are present within the *M. truncatula* genome (Dataset S3 at Dryad). The circadian regulator *LHY* peaks at dawn. qPCR analysis confirmed this observation, and showed that temporal patterns of *LHY* expression were similar in roots and nodules, with a slight phase delay in roots, but preceded expression in shoots by several hours in constant light (Supplementary Fig. S3). *PRR5* homologues were expressed in consecutive waves, with *PRR5c* and *PRR5a* peaking 3 h and 9 h after dawn, and *PRR7a* and *TOC1* peaking at dusk (Supplementary Fig. S4). Evening complex genes *LUX*, *ELF3*, and *ELF4* were expressed at dusk, whereas *GI*, which plays a role in light-dependent turnover of the TOC1 protein, was expressed in the late afternoon (Supplementary Fig. S4). The temporal expression patterns of these circadian clock-associated genes, including *LHY*, were consistent with those in our root dataset (Dataset S2 at Dryad), leaves of *A. thaliana* (Michael *et al.*, 2008; Pokhilko *et al.*, 2012), rice and poplar (Filichkin *et al.*, 2011), and the legume soybean (Marcolino-Gomes *et al.*, 2014), suggesting that circadian clock mechanisms are largely conserved between these plant species, and between roots, nodules, and plant leaves. We observe a delay in the phase of some key circadian clock genes in nodules with respect to roots (e.g. *MtLHY*, *MtTOC1*, and *MtPRR7*) but overall the phase is not significantly different (Fig. 1D).

Hierarchical clustering to assess patterns of expression identified three broad groups of rhythmically expressed transcripts (clusters) for both nodules and roots (Fig. 1B, C; Supplementary Fig. S2), and we found that >80% of the transcripts in both tissues peak during morning or evening. The morning cluster (gold) contains 1813 transcripts (64%) in nodules and 424 (47%) in roots, the evening cluster (red) has 520 transcripts (18%) in nodules and 322 (36%) in roots, and the night cluster (blue) is comprised of 499 transcripts (18%) in nodules and 158 (17%) in roots (Fig. 1B, C). This contrasted with previous observations in *A. thaliana* whole plants that the majority of cycling genes peak either before dawn or dusk (Michael *et al.*, 2008). Compared with roots, we found a much larger number of transcripts and a broader range of processes annotated as related to metabolism in nodules, which might reflect complex regulation of these processes by the circadian clock in nodules (Dataset S4 at Dryad).

### Rhythmic coordination of nodule and root metabolism

To determine which processes are rhythmic in belowground tissues and understand the importance of the biological processes in each cluster in both tissues, we analysed gene descriptions (obtained from Phytomine in Phytozome) as well as Gene Ontology (GO) term, biological pathway, and protein domain enrichment. For the purpose of reflecting those processes that are truly orchestrated by the circadian clock in a biologically significant manner, we plotted the phase of the transcripts associated with a specific pathway, GO term, or protein domain enrichment and selected those that peaked whithin a narrow window; Dataset S4 at Dryad.

The nodule morning cluster was enriched for genes annotated to ureide biosynthesis, gluconeogenesis, and spermine biosynthesis pathways, which are all related to N metabolism. Genes in the spermine biosynthesis pathway were also enriched in the root morning cluster (Fig. 1C). These morning processes were largely related to N metabolism in nodules, and the morning cluster has many genes associated with glutamate metabolism, amino acids, and nitrate/nitrite transport including glutamine synthetase (Medtr3g065250), which catalyses the first step of N assimilation. Ureides are the main long-distance transport forms of N from nodules to the shoot and are moved up the xylem vessels to the leaf tissue where then they are used as an N source (Liu *et al.*, 2018). Although indeterminate nodule-forming legumes including *M. truncatula* have been classified as amide type (rather than ureide type), detection of ureide pathway-related genes suggests that this part of the metabolism still occurs in these legumes as a response to N fixation (Sprent, 2007). In *A. thaliana*, genes associated with the isoflavonoid pathway are found to be expressed in the morning (Harmer *et al.*, 2000) and similar encoding genes are found to be expressed in the nodule morning cluster, including the rate-limiting enzyme phenylalanine ammonia-lyase (PAL). This cluster was also enriched for genes involved in elongation growth, including the transcription factor phytochrome-interacting factor 4 gene (*PIF4*, Medtr3g449770), which promotes auxin biosynthesis in *A. thaliana* (Franklin *et al.*, 2011), and eight Walls Are Thin1 (WAT)1-related genes encoding glycoside hydrolases, all related to cell wall biosynthesis and flavonoid biosynthesis (Ranocha *et al.*, 2010). These processes are typically linked to either defence or growth.

Interestingly, six key symbiotic genes, *NIN*, *RPG*, *CRE1*, *DMI2* (*SYMRK/NORK*), *DMI3* (*CCaMK*), and *KIN5* are rhythmically expressed in the morning cluster, peaking at ~24 h. *CRE1*, *DMI2*, and *KIN5* are linked to early symbiotic interaction, *NIN* is considered a ‘master coordinator’ of nodule development, *DMI3* is known to be essential for the initiation of symbiotic gene expression, and *RPG* is linked to rhizobial polar growth during nodulation; all reviewed in Roy *et al.* (2020). The clock could act to coordinate the expression of these key regulators.

The nodule evening cluster contained genes associated with glycogen degradation and late nodulins, and genes associated with glycogen degradation were also present in the nodule night cluster. (Fig. 1B). With gluconeogeneis peaking in the morning and glycogen degradation peaking in the evening and night clusters, there seems to be a C cycling process in nodules that might be governed by the demands of the N-fixing symbionts in this tissue. This C cycle in nodules is consistent with the pattern of starch accumulation and degradation in *A. thaliana* plants, where starch accumulates during the light period, then is utilized to support growth during the night (Graf *et al.*, 2010). There is also an over-representation of the ‘late nodulin domain’ which here is annotated to nodule-specific cysteine-rich peptides (NCRs) which are known to play an important role in controlling rhizobial activities (Roy *et al.*, 2020). The majority of rhythmically transcribed NCRs peak in the evening cluster, suggesting a link between nodulation and the circadian clock that could involve these regulatory genes.

Together, these results indicate that key aspects of nodule function, including C cycling, N assimilation, and N transport, occur rhythmically under the control of the circadian clock, and suggest that appropriate temporal coordination of these processes may be important for optimal nodule function. With complex C and N compounds being synthesized in the morning, findings of genes annotated to these functions suggest that this might be an anabolic time for nodules, with C catabolism occuring in the evening and night and N fixation in the bacteroids during the evening–night period, as was observed in pea many years ago (Minchin and Pate, 1974).

### *Loss of LHY* function disrupts circadian rhythms and impairs nodulation in *M. truncatula*

**Fig. 2.**
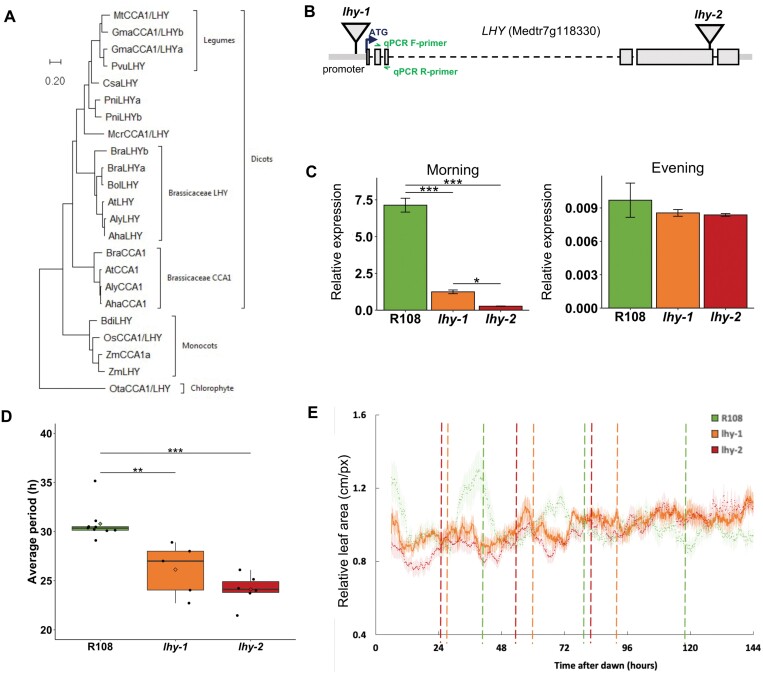
Loss of *M. truncatula LHY* expression affects plant rhythmicity and nodulation. (A) Phylogenetic analysis of CCA1/LHY homologues in Plantae and Chlorophyte. *MtLHY* (Medtr7g118330) shares 36.6% identity at the amino acid level with *AtCCA1* (At2g46830) and 44.2% identity at the amino acid level with *AtLHY* (At1g01060); Aha, *Arabidopsis helleri*; Aly, *Arabidopsis lyrata*; At, *Arabidopis thaliana;* Bdi, *Brachypodium distachyon*; Bol, *Brassica oleracea*; Bra, *Brassica rapa*; Csa, *Castanea sativa*; Gma, *Glycine max*; Mcr, *Mesembryanthemum crystallinum*; Mt, *Medicago truncatula*; Os, *Oryza sativa*; Ot, *Ostreococcus tauri*; Pni, *Populus nigra*; Pvu, *Phaseolus vulgaris*; Zm, *Zea mays*; see Dataset S5 at Dryad for gene IDs. (B) Location of *lhy-1* and *lhy-2* insertions in the *LHY* gene. (C) Relative expression of *MtLHY* in wild-type R108 (green), *lhy-1* (orange), and *lhy-2* (red) mutant plant leaves in the morning and evening periods. (D) Period of leaf movement rhythms for each genotype inferred from experimental data using the FFT-NLLS algorithm in BioDare2; for data see Dataset S5 at Dryad; black circles indicate individual plants, the diamond indicates mean period. (E) Disrupted leaf movement rhythms in *lhy* mutants in constant light; dashed vertical lines indicate the mean period for each genotype.

In order to investigate circadian regulation of nodulation, we tested the importance of *MtLHY* (Fig. 2A) in the regulation of nodulation by isolating and characterizing two mutants with reduced *MtLHY* expression from the Noble collection of retrotransposon insertion (*Tnt1*) lines (Fig. 2B). The *lhy-1* mutant contained an insertion upstream of the translational start site, probably in the promoter of *LHY*, and exhibited strongly reduced expression of the *LHY* transcript (20% of wild-type levels; Fig. 2C). The second mutant, *lhy-2*, had an insertion at the end of the second-last exon and negligible expression of the *LHY* transcript (Fig. 2C). We examined the rhythmicity of our *lhy* mutants by measuring the rhythmic opening and closing of the first true leaf. Plants were grown for 10 d under 12L:12D cycles then imaged over 7 d in constant light. The wild-type R108 showed sustained rhythmicity for over a week in constant light, with a period length of ~30 h. In contrast, both mutant alleles exhibited leaf movement rhythms with shorter free-running periods (~25 h) and a much lower amplitude, then became arrhythmic after 120 h (Fig. 2D, E). Leaf opening in the mutants occurred 6 h early on the first day following transfer to constant light, indicating that both mutations resulted in a large phase advance in constant light (Fig. 2E). These results demonstrated that loss of *LHY* function alters the function of the circadian clock in *M. truncatula*.

**Fig. 3.**
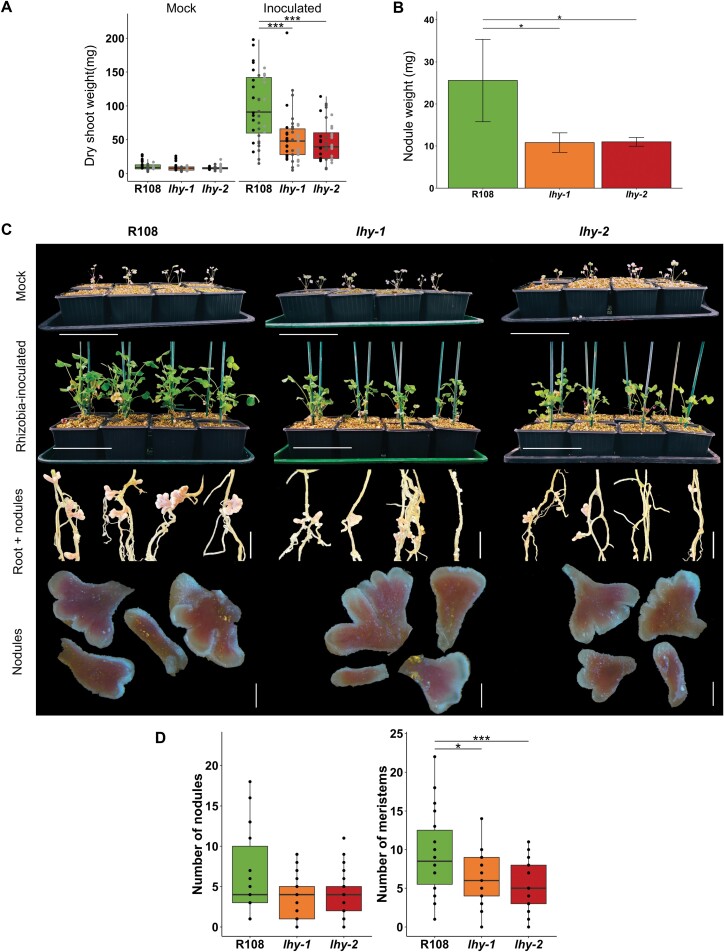
Loss of *M. truncatula LHY* expression affects nodulation under 16L:8D cycles. (A) Plants have a similar dry aboveground weight phenotype in the absence of rhizobial inoculation, but with inoculation the *lhy* mutants have reduced dry weight; boxplots with individual replicate data; *n*=24; ∗∗∗*P*<0.005. (B) Reduced nodule weight for *lhy-1* and *lhy-2* compared with the wild type R108; *n*=24; ∗*P*<0.05. (C) Images of 6-week-old mock- (top row) or *Sinorhizobium meliloti* WSM1022-inoculated plants grown in perlite–vermiculate pots showing reduced growth (second row) and less ramified nodules in the *lhy* mutants (lower rows). Scale bars for the upper two rows=10cm, middle row 1cm, lower row 0.1cm. (D) Nodule and nodule meristem lobe counts from inoculated plants; *n*=21–29; ∗*P*<0.05, ∗∗∗*P*<0.005; see Dataset S5 at Dryad for all values and analyses.

In order to assess the effect of *lhy* mutations on nodulation, we inoculated three biological replicates of *lhy-1*, *lhy-2*, and R108 seedlings with the high-efficiency rhizobial symbiont *S. meliloti* WSM1022. When grown under 16L:8D, both mutants had lower nodule weight and lower dry shoot weight than the wild type (Fig. 3A–C; Dataset S5 at Dryad). Interestingly, our data show that *lhy* dry weight is similar to that of the wild type when not inoculated with rhizobia, but reduced when plants are inoculated with rhizobia, suggesting that the reduced weight in the mutant is largely due to disrupted nodulation (Fig. 3A–C). Whilst individual nodule numbers are not different in the mutants compared with the wild type (Fig. 3D), we found a significant reduction in the overall number of branched nodule meristems in the *lhy* mutants (Fig 3D). The less ramified nodules in the *lhy* mutants might contribute to a less efficient N fixation process, indicating that normal function of *LHY* is required for optimal nodulation as well as plant growth.

### Regulation of gene expression via the evening element

In order to ask if there was a link between the circadian clock and regulation of nodule activity, we analysed nodule-specific transcripts with a regulatory role in nodulation. We found that the transcripts of 45 NCRs were rhythmic and there was an enrichment of NCR transcripts within the evening and night clusters (*P*=5.67e^–14^ and *P*=0.02 respectively) with 12 NCRs in the morning cluster 1, 22 NCRs in the evening cluster 2, and 11 NCRs in the late-night cluster 3 (Dataset S6 at Dryad). Their regulation may therefore be part of the mechanism by which the plant circadian clock impacts on nodulation.

**Fig. 4.**
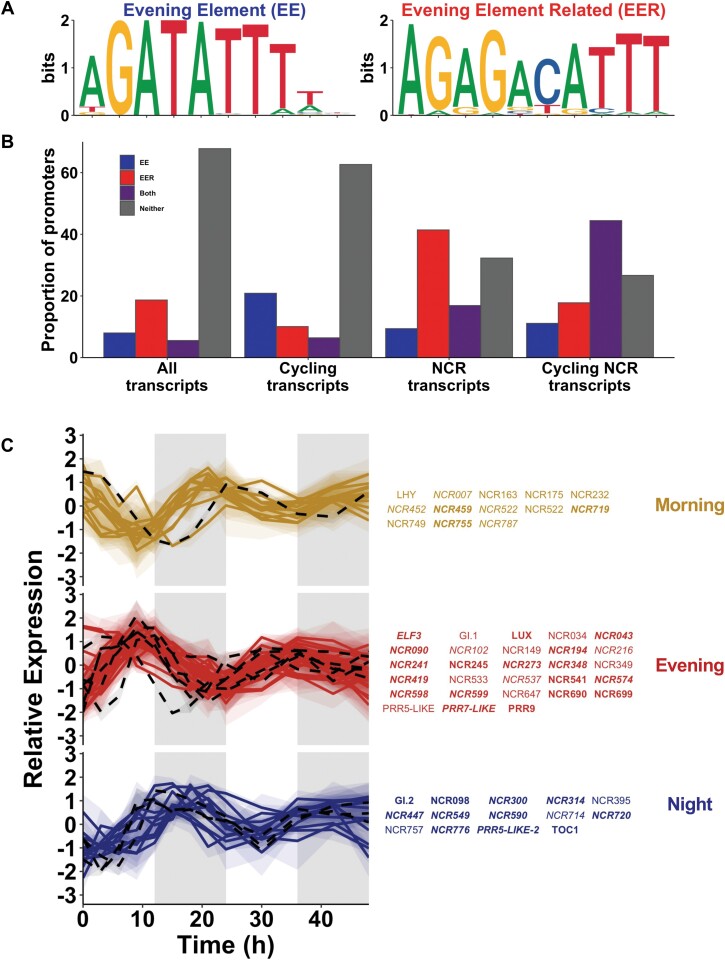
The evening element is enriched in the promoters of oscillating NCRs. (A) Motif comparison of the *A. thaliana* LHY/CCA1-binding site (EE) and *M. truncatula* evening element-related (EER) putative motif. (B) Proportion of 500 bp promoters containing matches to either the EE, EER, or both motifs. (C) Expression profiles of circadian clock genes (black, black dashed line in the graphs) and NCR genes (coloured, coloured solid lines in the graph) within each cluster. The average and range of each group of genes are indicated with lines and a cloud, respectively. NCRs with the EE motif in their promoters are indicated in bold, EER in italic, and both motifs in bold and italic. See Datasets S6 and S7 at Dryad for all values and analyses.

In *A. thaliana*, the circadian clock-associated proteins CCA1 and LHY bind a promoter motif with canonical sequence AGATATTT (Fig. 4A), known as the EE (Harmer *et al.*, 2000, Nagel *et al.*, 2015; Kamioka *et al.*, 2016; Adams *et al.*, 2018). To assess the importance of LHY in NCR expression, we analysed the upstream regions of rhythmic NCRs for the EE PWM. The EE was found to be significantly enriched in rhythmically expressed nodule transcripts compared with all rhythmically expressed transcripts (*P*=2.21e^–13^), and 5.4-fold enriched in rhythmically expressed NCR promoters compared with all rhythmic promoters (*P*=6.04e^–6^; Fig. 4B, C). The EE is found in 24.2% of all *M. truncatula* promoters, and is specifically enriched in rhythmic NCR promoters, with 26.0% of the promoters of a list of 743 transcripts compiled from Montiel *et al.* (2017) and de Bang *et al.* (2017). Almost all promoters that contain the EE had a single occurrence of the motif, with just three promoters having two occurrences (Dataset S6 at Dryad). A total of 86 NCR promoters contained an EE but did not oscillate in our experiment (Dataset S6 at Dryad). This is consistent with previous observations that a large proportion of CCA1 and LHY regulatory targets do not exhibit rhythmic expression under any observed condition (Nagel *et al.*, 2015; Adams *et al.*, 2018). Expression of these NCRs may cycle at other stages of nodule development or in specific nodule cell types.

To determine if other motifs may be enriched within NCR promoters, we carried out a *de novo* motif analysis in promoters of NCRs with rhythmic expression. This identified three over-represented motifs within 500 bp upstream of the transcriptional start sites, and within 200 bp of the TATA box. These ~12 bp motifs mapped into longer stretches of conserved sequences identified in a previous study (Nallu *et al.*, 2013), with motif 2 (AGA[T/C]ATTT, Supplementary Fig. S1) being highly similar to the *A. thaliana* EE. We also found an alternative version of the EE motif, which we call EER (Fig 4A), to be 3.6-fold and significantly enriched (*P*=5.19e^–147^), with 57.6% of rhythmic NCRs containing an EER motif compared with an abundance of 16.2% of all NCR promoters. Across all NCRs, the presence of the EE or of the EER motif was evenly distributed across the phylogeny, suggesting that they did not arise as part of a single lineage-specific expansion event (Supplementary Fig. S5). Consistent with the hypothesis that NCRs are regulated by the LHY transcriptional repressor, the majority of NCRs with an EE motif in their promoter peak in expression in the morning, when *LHY* transcription is low (Fig. 1A). We asked how rhythmic NCRs are expressed in the *lhy* mutants compared with R108 by measuring their expression at ZT1–2, when *LHY* expression would normally be high and thus NCR expression repressed. We found that NCRs with an EE are expressed 2% or 17% more highly in the *lhy-1* or *lhy-2* background compared with R108, than NCRs without an EE, suggesting that LHY-EE regulation is important for NCR repression in the morning (Dataset S5 at Dryad).

## Discussion

Many aspects of physiology, metabolism, and development exhibit circadian regulation across plants, animals, and some microbes. Thus, the circadian clock often influences the outcome of interactions between organisms (de Leone *et al.*, 2020). In plants, the oscillator mechanism of the circadian clock has been investigated at length in shoots (reviewed in McClung, 2019). In roots, it is known to be entrained by shoot-derived signals (Takahashi *et al.*, 2015). Despite the agricultural importance of the beneficial legume–microbe interaction of nodulation, very little is known about the impact of the plant circadian clock on this N-fixing symbiosis. In common bean (*Phaseolus vulgaris*), changes in expression levels of circadian clock-associated genes were detected in the early stages of symbiosis, suggesting that the function of the root circadian clock was adjusted in response to infection by rhizobial strains (Dalla Via *et al.*, 2015).

In order to obtain clues to the mechanisms by which the circadian clock might affect nodulation, we asked which processes are rhythmic in nodules using time course transcriptome analysis. Overall, ~5% of the transcriptome showed rhythmic expression in constant light in nodules and ~1% in roots. The proportion of oscillating transcripts in nodules is comparable with the 6% observed under free-running conditions in Arabidopsis lateral roots (Voß *et al.*, 2015). As no other circadian transcriptome data are available for plant roots or for *M. truncatula*, it is unclear whether this reflects a species difference or a root versus shoot difference. For genes that are rhythmic, we found that the temporal pattern of expression of circadian clock-associated genes in *M. truncatula* nodules is consistent with that observed in other plant species and in other organs, suggesting that the molecular mechanism of the central oscillator is largely conserved. However, a delay in peak times for some key circadian clock genes is observed in nodules with respect to roots, suggesting that the nodule clock might be a slave from that of the root in *M. truncatula*, as was found for the root with respect to the shoot in *A. thaliana* (James *et al.*, 2008).

While *LHY* and *CCA1* are closely related and have largely redundant functions in *A. thaliana*, the *M. truncatula* genome contains a single orthologue of these proteins. Loss of function of both genes is required to disrupt free-running rhythmicity in *A. thaliana* (Mizoguchi *et al.*, 2002), but in *M. truncatula* we show that loss of function of *MtLHY* leads to shorter period rhythms of leaf movements and gradual arrhythmia in constant light. We also show that loss of function of *LHY* results in reduced nodulation (Fig. 3B–D). Plant dry weight was reduced in *lhy* mutants that were inoculated with *S. meliloti* (Fig. 3A), but not in uninoculated plants, suggesting that reduced biomass might be caused by disrupted N fixation. The LHY-binding site, also known as the EE, was over-represented in the promoters of *M. truncatula* nodule-expressed genes that peaked in expression in the evening. This was consistent with a role for the cognate transcription factor MtLHY in driving rhythmic gene expression. These findings suggest that MtLHY plays a similar role to its *A. thaliana* orthologue and also acts as a core component of the nodule central oscillator. Our analysis of *lhy* is consistent with a previous report showing that *MtLHY* is expressed in a diurnal fashion (Kong *et al.*, 2020). This work also reported reduced nodulation in two different *Tnt1* lines (NF6569 and NF16126) having insertions at different position in the genome; therefore, it is unclear if this was a direct *LHY* effect.

We used transcriptomic analysis to ask how processes were regulated in nodules over time, enabling coordinated waves of processes to be discerned (Fig. 1B–D). Expression of genes associated with carbon catabolism was observed in the evening–night period, as previously described in *A. thaliana* (Harmer *et al.*, 2000). This was followed by expression of genes associated with ureide biosynthesis in the morning. Ureides are the main long-distance transport forms of organic N in legumes, and their production in the late subjective night suggests that N fixation in symbiosomes occurs during the night, deriving its energy from C catabolism. In support of this hypothesis, we also find transmembrane amino acid transporters in the late night/dawn gene clusters.

Genes associated with isoflavonoid biosynthesis peaked around dawn, as previously described in other plants such as Ginkgo (Ni *et al.*, 2018) and *A. thaliana* (Harmer *et al.*, 2000). Isoflavonoids are polycyclic compounds that belong to the wider group of phytoalexins that are synthesized by many plants, and many have antimicrobial activities. In legumes, a wide range of isoflavonoid compounds have been described, with the composition mix being different depending on the species (Dakora and Phillips, 1996). Some of these isoflavonoids actually initiate the plant–symbiont molecular dialogue that leads to nodule formation, by inducing the expression of *nod* genes in rhizobia (Peters *et al.*, 1986). The circadian clock is known to regulate plant defence responses, and plants are typically more resistant to pathogen attacks at dawn (Bhardwaj *et al.*, 2011; Shin *et al.*, 2012; Lu *et al.*, 2017). Production of flavonoids at dawn may contribute to this gating mechanism, to control entry of microbes into plant roots while attracting rhizobial symbionts. However, flavonoids are thought to have a role beyond initial recruitment of rhizobia, since they are mostly produced in the nodule infection zone, where bacteroids become fully elongated and start to express N fixation genes (Chen *et al.*, 2015). In mature nodules, isoflavonoids have been suggested to play a role in maintaining a homogeneous rhizobial population (Liu and Murray, 2016). Since expression of genes associated with spermine biosynthesis (also in roots) and cell wall metabolism peaks in the morning, rhythmic production of flavonoids could act to coordinate nodule cell expansion with bacteroid proliferation at dawn. The presence of six key symbiotic genes in our morning cluster, all linked to several processes such as bacterial infection, rhizobial polar growth, nodule growth, or development, suggests that many complex processes that happen during nodule growth and infection are tightly orchestrated by the nodule circadian clock in *M. truncatula*. Since the peak expression of these genes is around dawn, it may suggest that dawn might be key for nodule cell expansion and coordination of infection, and the evening/night key for nitrogen fixation.

Our transcriptomic analysis also revealed the rhythmic expression of a subset of NCRs, with the majority peaking in the evening (Fig. 4C). This large family of peptides is thought to control bacterial differentiation within the nodule, but there is evidence for functional differentiation of NCRs, as different NCRs can have either pro-symbiotic or anti-symbiotic properties (Wang *et al.*, 2017; Yang *et al.*, 2017), and bacterial elongation and activity in nodules can vary depending on the particular suite of NCRs present in the plant host (Montiel *et al.*, 2017). The observation that a subset of NCRs is expressed rhythmically in nodules suggests a function to synchronize bacterial activity with the rhythms of the plant host and provides further evidence for functional differentiation of this group of peptides. Previous studies of NCR promoters identified long stretches of conserved sequence which included putative regulatory motifs such as an ID1-binding site, an auxin response factor (ARF)-binding site, a DOF protein-binding site, and MADS transcription factor-binding sites (Nallu *et al.*, 2013). Here we show that the EE motif is over-represented within NCR promoters (Fig. 4B), suggesting direct repression by the MtLHY circadian clock protein in the morning. This explains the temporal expression pattern of the majority of NCRs, peaking in the evening in cluster 3. Some NCRs peaking earlier or later in clusters 2 and 4 that contained EEs in their promoters are also likely to be regulated by MtLHY in combination with other rhythmic transcription factors. A related motif named EE-related or EER was also identified, which was not associated with expression at specific times of the day but was over-represented in NCR promoters, and thus may be associated with nodule-specific gene expression. This motif was also present within one of the stretches of conserved sequence previously identified in NCR promoters (Nallu *et al.*, 2013). The EE sequence (AGATATTT) lies in the same region within this conserved sequence, but was not uncovered in that previous research, probably due to the EER sequence variant (AGACATTT) being present at a high frequency. The presence of a cytosine in the EER motif is interesting because it could be associated with epigenetic regulation of expression (O’Malley *et al.*, 2016).

The coordination of nodule growth with bacterial differentiation and N fixation in indeterminate legume nodules is a well-orchestrated process. Our results suggest that rhythmic expression of NCR peptides under the control of the plant circadian clock plays a vital role in the establishment of successful symbiotic interactions. Many crops have lost their photoperiodic responses as part of domestication, because this was essential for cultivation at a broad range of latitudes and, in many cases, this happened through disruption of the circadian clock (Nakamichi, 2015). For example, circadian clock components have been modified during the soybean domestication process (reviewed in Li and Lam, 2020). It is therefore crucial to understand how it affects the host–symbiont interaction so we can avoid breeding against the efficiency of the N fixation process. Moreover, the possibility of modulating specific downstream pathways such as rhythmic NCR peptides may enable optimization of nodulation whilst avoiding undesirable plant circadian clock-related side effects. By identifying a mechanism that links control of plant growth and development with that of its symbiotic partner, our work opens up a new field of investigation for understanding how the rhizobial activity is regulated by the plant.

## Supplementary data

The following supplementary data are available at *JXB* online.

Fig. S1. Promoter landscape of the NCRs.

Fig. S2. Heatmaps of overall expression changes over time in nodules and roots.

Fig. S3. *LHY* is rhythmically expressed in nodules.

Fig. S4. Oscillation of circadian clock genes over the nodule time course.

Fig. S5. Phylogenetic tree of NCR promoter sequences shows high levels of similarity across the family.

erab526_suppl_Supplementary_Figures_S1-S5

## Data Availability

The raw RNA-seq data that support the findings of this study are openly available in the NCBI SRA database (PRJNA634620). The datasets associated with this paper are openly available at the Dryad Digital Repository https://doi.org/10.5061/dryad.9s4mw6mgg; Achom *et al.*, 2022).
