# Supplementary material for: Plant circadian clock control of Medicago truncatula nodulation via regulation of nodule cysteine-rich peptides
Source: J Exp Bot. 2021 Dec 1;73(7):2142–56. doi: 10.1093/jxb/erab526 (PMC8982390; doi:10.1093/jxb/erab526)
Supplement: erab526_suppl_Supplementary_Figures_S1-S5 [file erab526_suppl_supplementary_figures_s1-s5.pdf]

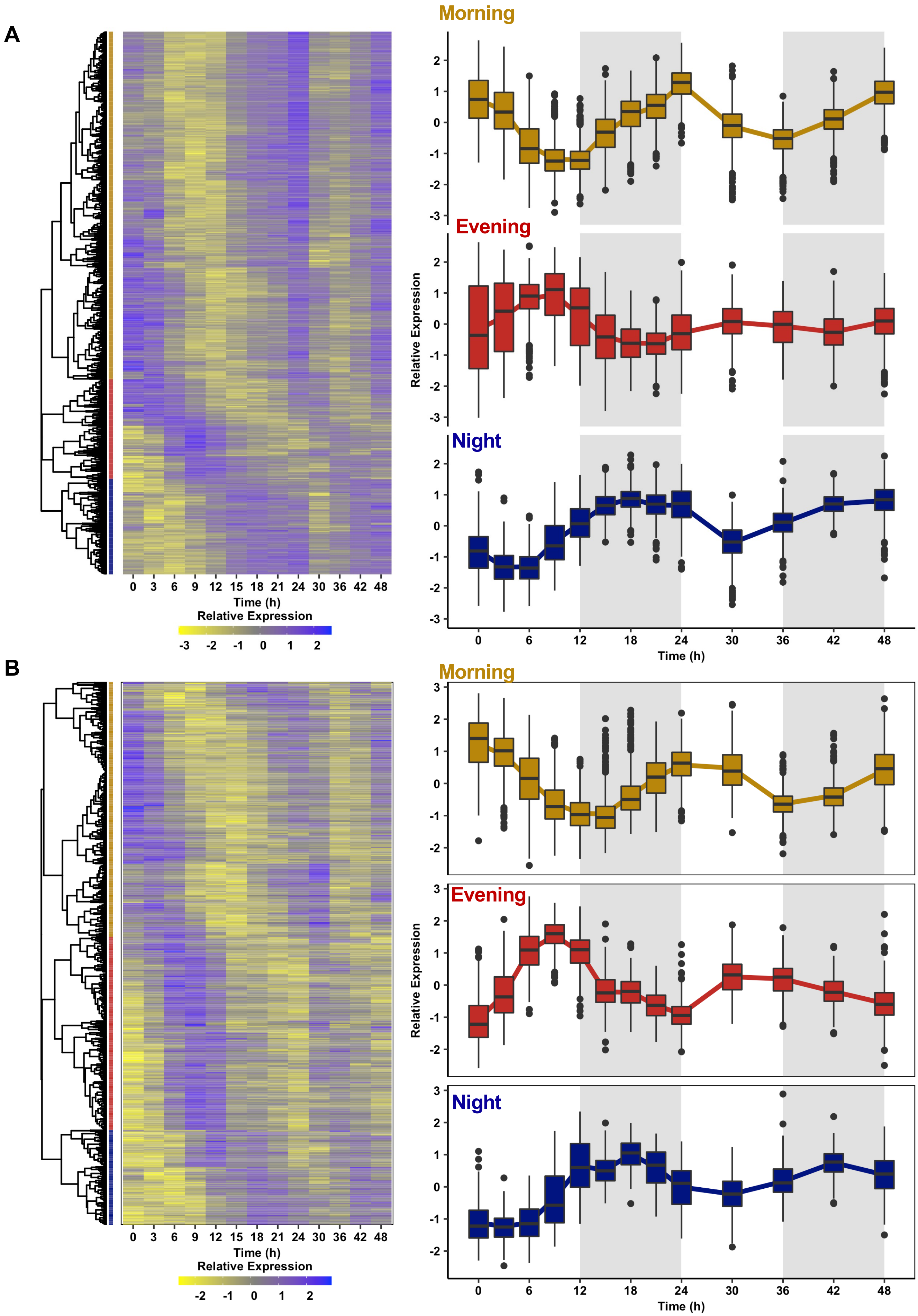

Figure S1. Heatmaps of overall expression changes over time in nodules and roots. Heatmaps of relative expression values of the (A) 2,832 and (B) 904 transcripts with a rhythmic pattern in nodules and roots respectively ($p<0.05$); see Supplementary Datasets S1-S2.

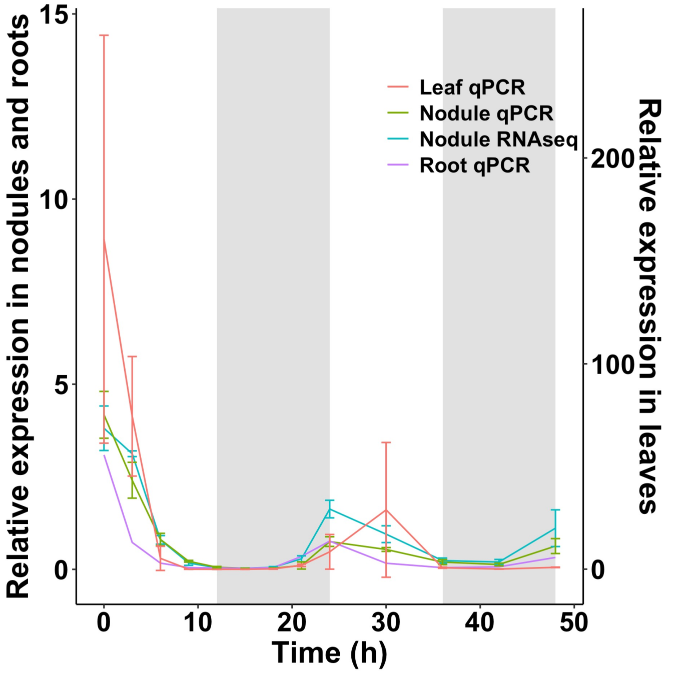

Figure S2. *LHY* is rhythmically expressed in nodules. *LHY* expression exhibits free-running rhythmicity with a dawn peak of expression, consistent with the presence of a functional circadian clock in nodules and roots. Expression measured using qPCR (normalised to beta-tubulin) in leaves (red), nodules (green) and roots (purple). Expression measurement in nodules using qPCR (green) corroborates the measurement using RNAseq (blue). White and grey background represents presumptive day and night; error bars indicate standard deviation of the mean (n=3 biological replicates). See Supplementary Dataset S3 for all data values.

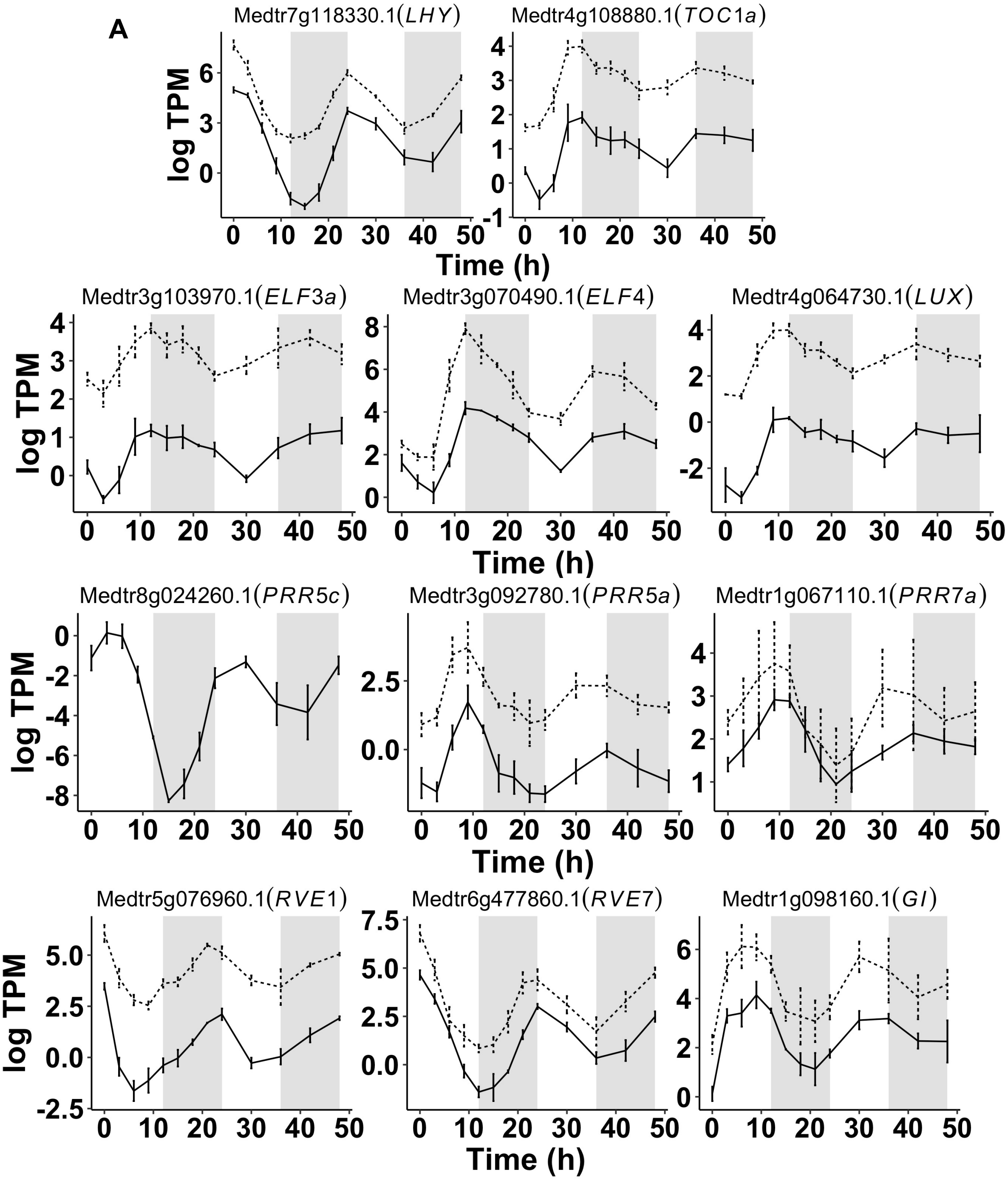

Figure S3. Oscillation of circadian clock genes over nodule timecourse. (A) The core oscillator: LHY/CCA1 and TOC1 form the core oscillatory loop via a negative feedback mechanism. (B) The Evening Complex (EC) is formed of three proteins, ELF3, ELF4 and LUX, whose expression peaks in the evening; (C) Pseudo-response regulators (PRRs) are homologous to TOC1 and expressed mainly during day-time to form additional loops. (D) RVEs and Gigantea (GI) synchronize the central clock with output pathways. White and grey background represents presumptive day and night (although plants were transferred from 12/12 hours dark/light to constant light before sampling); error bars indicate standard deviation of the mean (n=3 biological replicates, except for 15h and 21h where n=2); dashed lines indicate expression in roots, solid lines in nodules. See Supplementary Dataset S3 for all data values.

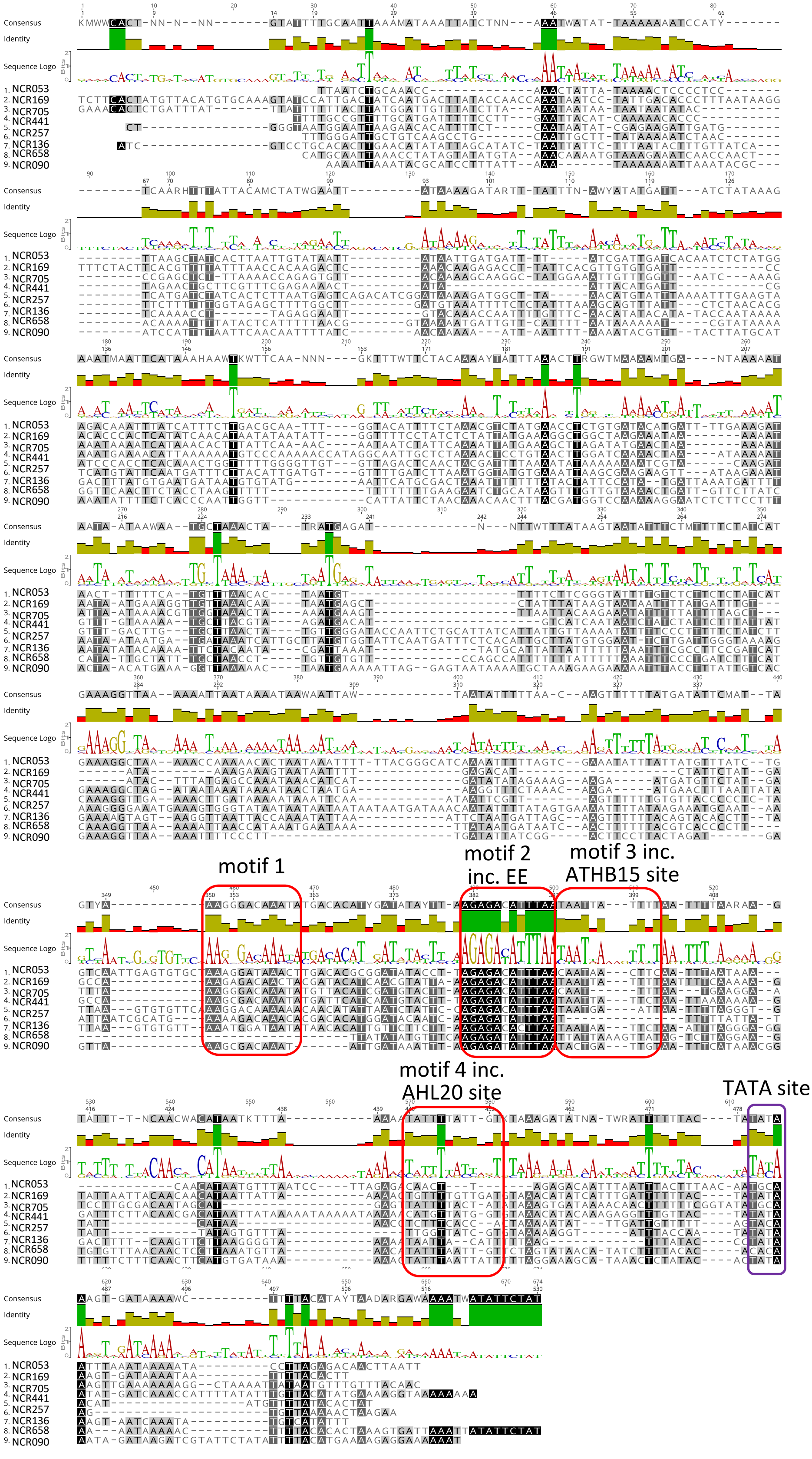

Figure S4: Promoter landscape of the NCRs Multiple Sequence alignment (MSA) visualisation of the conserved motifs in nine NCR promoters (of 166 that have temporal expression variation) that best represents the five conserved regions (red boxes) using Genious 1102. The visualization was performed after aligning 500bp upstream promoters using the EBI tool MAFFT (see Methods), aligned with open gap and gap extension penalty due to the diverse nature of the NCR sequences (Branca et al., 2011)(Mount, 2008). Conserved motifs were selected based on bits size (range from 0-2), positional bias (p-value<0.05) and with an E-value< 0.001. The evening element (EE) was found within the conserved region that we labelled 'motif 2', there is some homology between the binding sites of ATHB15/ATHB16 and motif 3, and with the binding site of AHL20 and motif 4. *de novo* identified motif 1 has no known transcriptional regulator. The TATA binding box is indicated within a purple box. See Supplementary Dataset S7 for enrichment statistical analysis.

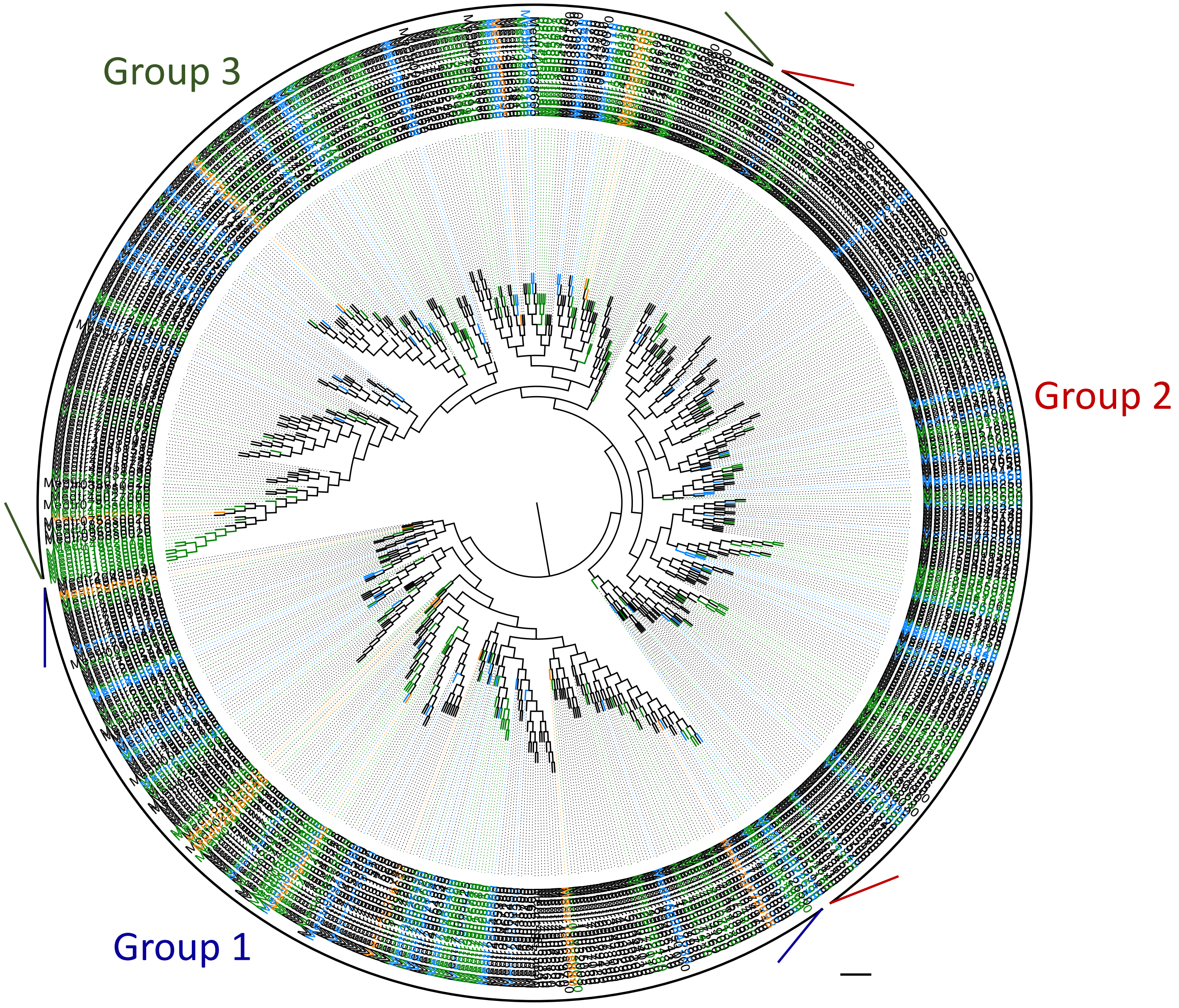

Figure S5. Phylogenetic tree of NCR promoter sequences shows high levels of similarity across the family. Polar visualisation of the promoters of 700 NCRs that all have promoter regions of 500bp, excepting three scaffolds (Medtr0538s0010, Medtr1886s0010, Medtr0753s0010). Blue colour denotes NCRs with the evening element, EE (AGATATTT) in their promoter region, green colour with the alternative motif AGACATTT and orange colour denotes NCRs with both the EE and alternative motif. The NCR promoters can be broadly divided into three groups (denoted 1,2,3 from the root outwards) in which there are NCRs with the EE or alternative EE. Promoters with both the EE and the alternative EE (orange) are only present in groups 1 and 3. The tree represents DNA maximum-likelihood phylogeny of NCR promoters with 100 bootstrapping and is rooted in the middle. Scale bar of 0.3 represents branch length, measured as the rate of nucleotide substitution per site.
